# Effect of sodium ferulate on delayed rectifier K^+^ currents in PC12 cells

**DOI:** 10.3892/etm.2014.1806

**Published:** 2014-06-24

**Authors:** WEI WANG, YUYUN WANG, CHUNLEI ZHANG, MENGMENG SUN, XIAOYIN ZHU

**Affiliations:** Basic Medical Department, School of Pharmacy, Yantai University, Yantai, Shandong 264005, P.R. China

**Keywords:** sodium ferulate, K^+^ channel, PC12 cells, automated patch-clamp

## Abstract

In order to investigate the effect of sodium ferulate (SF) on voltage-activated K^+^ channels, the delayed rectifier K^+^ current (Ik) in PC12 rat pheochromocytoma cells was recorded using the automated patch-clamp method. The results indicated that following the application of SF, the Ik in PC12 cells was significantly decreased in a concentration-dependent manner. The analysis of activation kinetic curves and inactivation kinetic curves of Ik showed that SF had an effect on the activation and inactivation kinetics. Following the application of 15.3 μM SF, the activation curve of the Ik of PC12 cells was shifted to positive potentials and the inactivation curve of the Ik of PC12 cells was shifted to negative potentials. This study revealed that the delayed rectifier K^+^ currents of PC12 cells were inhibited following SF treatment in a concentration-dependent manner. The mechanism may be associated with the delayed activation and enhanced inactivation of Ik-associated channels.

## Introduction

Sodium ferulate (SF), the sodium salt of ferulic acid, a type of organic acid extracted from the traditional Chinese herb Radix Angelicae Sinensis (Oliv.) Diels, is an approved drug used for the treatment of cardiovascular and cerebrovascular diseases in China. Pharmacological effects, including anti-oxidative and anti-inflammatory activity, inhibition of platelet aggregation and free radical-scavenging activity have been demonstrated (1,2). It has been reported that SF may decrease the pain transmitted during chronic neuropathic pain injury (3). Therefore, the present study investigated whether SF has an effect on ion channels. Ion channels of various types are associated with neuronal excitability since they are important physiological regulators of action potential shape, membrane potential and firing adaptation in excitable tissues (4,5). In particular, the delayed rectifier K^+^ current (Ik), which is representative of the voltage-gated K^+^ (Kv) channel, has been previously investigated in order to determine the anti-nociceptive mechanism (6,7) since it has an important role in cellular signaling processes, including membrane excitability and neurotransmitter release (8,9).

The patch-clamp technique is a state-of-the-art technology for the study of ion channels; however, patch clamping is a laborious process, which requires skilled and trained individuals. Recently, the automated patch-clamp technique has been developed, and research investigating cell channels has increased markedly. It allows for better qualification of hits and shortens the length of time required to study novel drugs (10,11). However, it is difficult to capture primary neurons in order to record the Ik for neuropathic pain studies since there are a large number of glial cells in the original generation of cultured cells, which increases the capture error. Therefore, undifferentiated PC12 cells, chromaffin-like cells derived from rat adrenal medulla that express the Ik and exhibit typical neuronal characteristics in form and function, are widely used as an *in vitro* model for neuron research (12,13).

In the present study, the effect of SF on the Ik through the Kv channel of neuronal PC12 cells was determined using an automated patch-clamp method. The results may provide data useful in explaining the mechanism of the analgesic effect of SF.

## Materials and methods

### Drugs and chemicals

SF [molecular formula, C_10_H_9_NaO_4_·2H_2_O; molecular weight, 252.20; CAS, 24276-84-4; high-performance liquid chromatography purity, >98%) was provided by Beijing SL Pharmaceutical Co., Ltd. (Beijing, China). N-2-hydroxyethylpiperazine-N′-2-ethanesulfonic acid (HEPES) and ethyleneglycol bis(β)-aminoethyl ether N,N,N′,N′-tetraacetic acid (EGTA) were obtained from Sigma-Aldrich (St. Louis, MO, USA). Fetal bovine serum (FBS), Dulbecco’s modified Eagle’s medium (DMEM) and heat-inactivated horse serum (HS) were purchased from Gibco (Grand Island, NY, USA). The other reagents were obtained from Sinopharm Chemical Reagent Co. Ltd. (Shanghai, China).

### Cell culture

Undifferentiated PC12 cells were purchased from the Type Culture Collection of the Chinese Academy of Sciences (Shanghai, China). The cells were maintained in DMEM, supplemented with 5% FBS, 10% HS, 100 U/ml penicillin, 2 mM glutamine and 100 mg/ml streptomycin, in the absence of a nerve growth factor (14). Cells were maintained on Petri dishes at 37°C, in a 5% CO_2_ humidified atmosphere. The culture medium was changed every 3–5 days and cells were split when necessary.

### Solutions

For the Ik recording, the composition of the standard external solution was as follows: 5 mmol/l KCl, 145 mmol/l NaCl, 2 mmol/l CaCl_2_, 1 mmol/l MgCl_2_, 10 mmol/l HEPES and 10 mmol/l D-glucose monohydrate, with the pH adjusted to 7.3 with NaOH. The internal solution was as follows: 1 mmol/l MgCl_2_, 135 mmol/l KCl, 1 mmol/l EGTA, 10 mmol/l HEPES, 1 mmol/l ATPNa_2_ and 10 mmol/l glucose, with the pH adjusted to 7.3 with KOH. The seal enhancer, which was used to assist stable seal formation at the seal formation step, was as follows: 3 mmol/l KCl, 80 mmol/l NaCl, 35 mmol/l CaCl_2_, 10 mmol/l HEPES and 10 mmol/l MgCl_2_, with the pH adjusted to 7.2 with NaOH.

### Automated patch-clamp current recording

The automated patch-clamp device (NPC-16 Patchliner; Nanion Technologies, Munich, Germany), with a low-pass filter (10 kHz), a 4-pole Bessel filter and EPC 10 Patch Clamp Amplifiers (HEKA Elektronik, Lambrecht/Pfalz, Germany), was used to record the whole-cell Ik. The patch solutions and cells were automatically acquired and they were added to the four wells in the microfabricated disposable chip. All the experiments were performed at room temperature (22–24°C).

In order to record the Ik, the holding potential was set to −70 mV. A 30 msec conditioning depolarization at −40 mV then followed in order to inactivate the Na^+^ channels. 10-mV step pulses (200 msec duration) between −60 and +40 mV were applied. Ik was evoked and did not decay in the 200-msec course. In order to indicate a delayed rectifier K^+^ current, the peak outward currents were blocked to 22.0±1.4% of the control values with 5 mM tetraethylammonium, as previously described for undifferentiated PC12 cells (15). For recording the effect of SF on Ik, four concentration of SF were used: 3.8, 7.7, 15.3 and 30.6 μM. Each concentration of SF was added once to the cells and maintained for ≥300 sec until currents reached equilibrium. In each group, five valid data cells were recorded (n=5). Peak amplitudes of the currents were measured. The normalized current was calculated using the formula: Normalized current = Idrug/I × 10 (where Idrug and I are peak amplitudes of Ik following and prior to the application of SF, respectively).

In order to record the activation kinetic curves of Ik, the cells were held at −70 mV (the holding potential), and the potassium currents were elicited with the application of 10 mV step pulses, ranging between −70 and +80 mV. A concentration of 15.3 μM SF was used to determine the effect of SF on the activation kinetic curves, since at this concentration Ik was inhibited ~50% (close to IC_50_). For the regression analysis of the activation kinetic curves of Ik, Ik was converted into conductance using the following equation as previously described by Wang *et al* (16): G = I/(V-Vrev), where G represents conductance, V represents the membrane potential, Vrev represents the reversal potential and I is the K^+^ current. The normalized conductance was shown using the following Boltzmann equation: G/Gmax = {1 + exp[(V1/2−V)/k>, where G/Gmax represents normalized conductance, V1/2 represents membrane potential at half-activation, and k represents slope factor.

In order to determine the inactivation kinetic curves of Ik, cells were held at −70 mV (the holding potential) and the potassium currents were elicited with a test pulse to +40 mV, following a prepulse of between −60 and +80 mV, in increments of 10 mV. The concentration of 15.3 μM was selected to record the effect of SF on the inactivation kinetic curves of Ik, as aforementioned. For the regression analysis of inactivation kinetic curves, the steady-state inactivation curves were shown by the following Boltzmann equation: I/Imax = {1 + exp [V1/2−V)/k]], where I/Imax represents the normalized currents, V1/2 represents the membrane potential at half-inactivation and k represents the slope factor.

Recordings were only performed from cells when the seal resistance was >1 GΩ. Five kHz was automatically obtained and set for the current recordings. When the currents reached equilibrium (≥200 sec), the extracellular solution was replaced with the extracellular solution containing drug using 40 μl solution via 4 pipette tips of the NPC-16 patch liner.

### Data analysis

Whole-cell data files were imported into Igor Pro (WaveMetrics, Inc., Portland, OR, USA). Results are presented as the mean ± standard error of the mean. Microsoft Excel (Microsoft Corporation, Redmond, WA, USA) and Sigmaplot (SPSS, Inc., Chicago, IL, USA) software were used for analysis or display. One-way analysis of variance and the Student’s t-test were used to compare the difference of drug effects. P<0.05 was considered to indicate a statistically significant difference.

## Results

### Effect of SF on the Ik of PC12 cells

**Figure 1:**
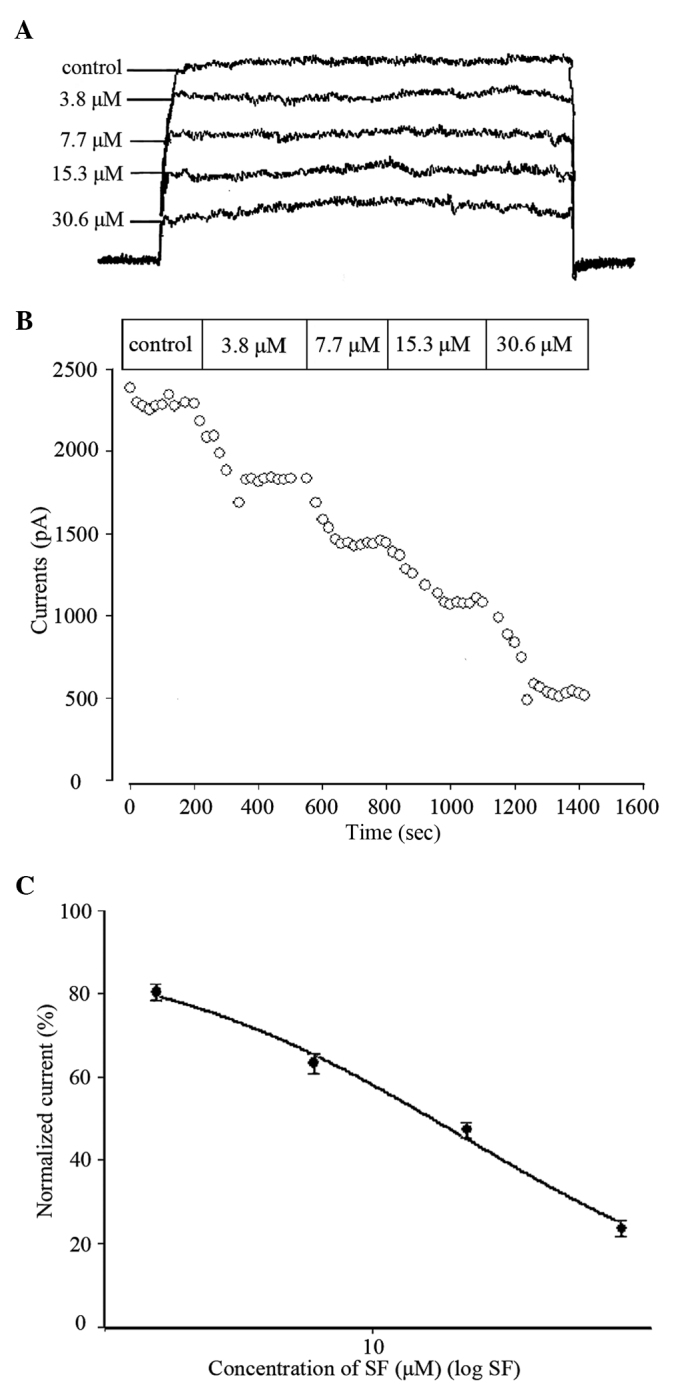
Effect of SF on the Ik in PC12 cells. Ik was recorded from PC12 cells using an automatic patch clamp. (A) The Ik in the absence (control) and presence of four concentrations of SF (3.8, 7.7, 15.3 and 30.6 μM). (B) The peak amplitudes of Ik were decreased with increasing SF concentrations. Four concentrations of SF were respectively applied to the patched cell in a cumulative manner in the representative time trace of Ik. (C) Concentration-normalized current following treatment with 3.8, 7.7, 15.3 and 30.6 μM SF. Each concentration of SF was applied once for ≥300s until currents reached equilibrium. SF, sodium ferulate; Ik, delayed rectifier K^+^ current.

The holding potential was set to −70 mV and a 30 msec conditioning depolarization to −40 mV followed (to inactivate the Na^+^ channel) and 10-mV step pulses (200 msec duration) between −60 mV and +40 mV were applied. As shown in Fig. 1, compared with the control, the peak amplitudes of Ik were decreased following the application of SF in a concentration-dependent manner. The half inhibitory concentration (IC_50_) and the Hill coefficient of SF were 14.9 and 0.87 μM, respectively.

### Effect of SF on the activation kinetics of Ik

**Figure 2:**
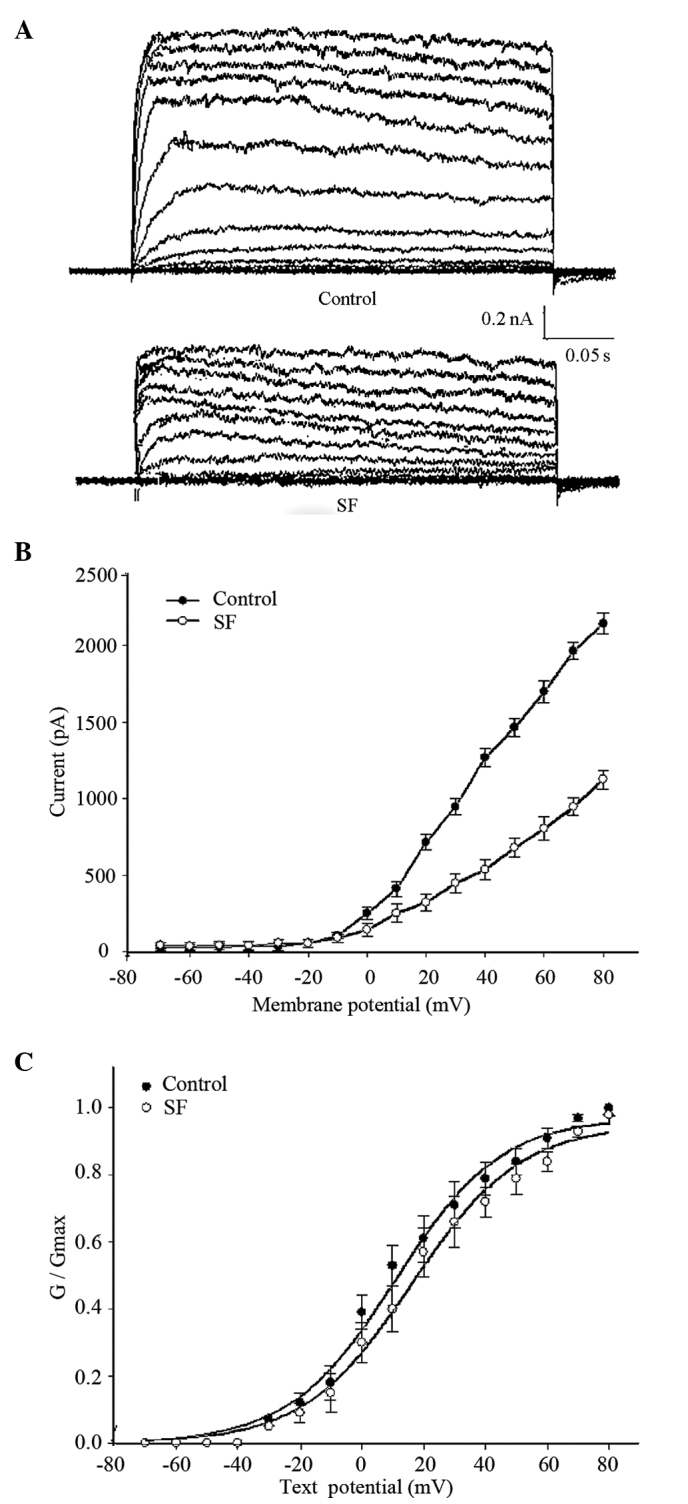
Effect of SF (15.3 μM) on the activation kinetics of Ik. (A) Cells were held at −70 mV, and currents were induced following step voltage pulses, ranging between −70 and +80 mV, in increments of 10 mV. Superimposed Ik responses to the application of step voltage pulses (in the absence of SF) and superimposed Ik responses to the application of step voltage pulses following the application of 15.3 μM SF. (B) The current-voltage (I-V) association of Ik in the absence (control) and following (SF) application of 15.3 μM SF. I-V curves were calculated by plotting peak current amplitude of Ik against the test potentials. (C) The steady-state activation kinetic curves for Ik in the absence (control) and following (SF) application of 15.3 μM SF. The data is presented as G/Gmax against the test potentials and fitted with a Boltzmann equation G/Gmax = {1 + exp[(V1/2−V)/k]. SF, sodium ferulate; Ik, delayed rectifier K^+^ current.

The effect of SF (15.3 μM) on the activation kinetics of Ik is shown in Fig. 2. The currents were induced during repeated sweeps to +80 mV from a holding potential of −70 mV, in increments of 10 mV. Following the application of 15.3 μM SF, the induced Ik was inhibited (Fig. 2A and B). The curves were fitted with the Boltzmann equation (r=0.997) and the values of V1/2 for activation of Ik changed from 11.2±1.8 mV (absence of SF) to 18.9±2.1 mV (n=5; P<0.01), and the slope factor k of the G/Gmax curves showed no significant difference prior to and following the drug treatment (from 17.3±1.6 to 17.8±1.4 mV; n=5, P>0.05; Fig. 2C). Compared with the control, SF shifted the activation kinetic curve to a positive potential and had no effect on the slope factor.

### Effect of SF on the inactivation kinetics of Ik

**Figure 3:**
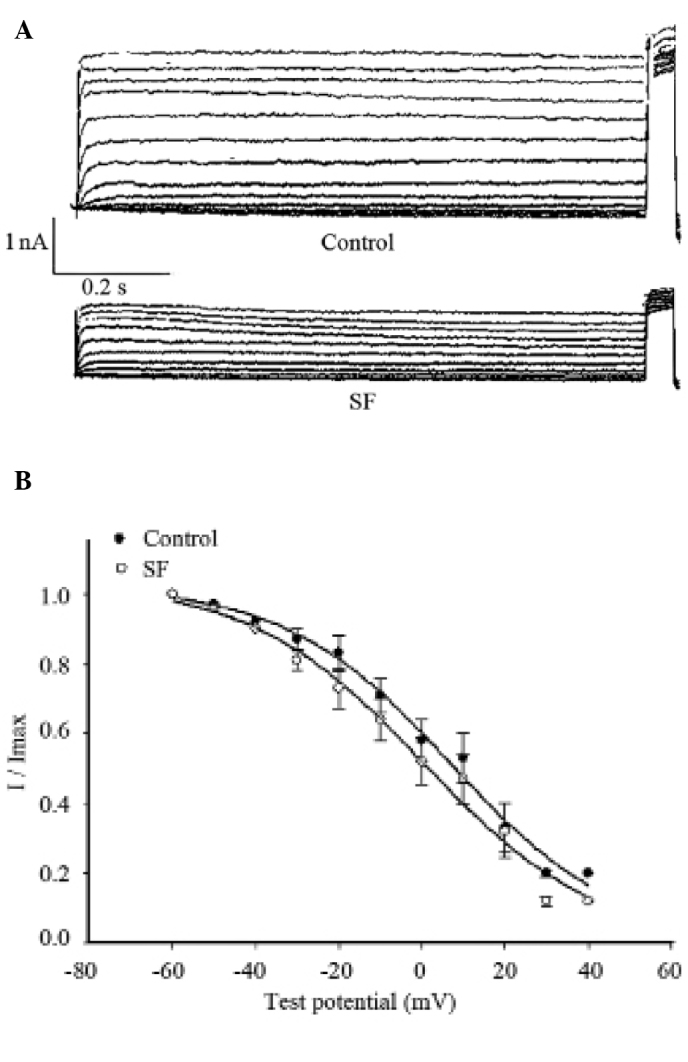
Effect of SF (15.3 μM) on inactivation kinetics of Ik. (A) Cells were held at −70 mV and the currents were elicited with a test pulse to +40 mV, during repeated sweeps to +80 mV from a prepulse of −60 mV, in increments of 10 mV. Superimposed Ik responses to the application of step voltage pulses (in the absence of SF) and following application of 15.3 μM SF. (B) Ik steady-state inactivation kinetic curves in the absence (control) and presence (SF) of 15.3 μM SF. The data was shown by normalizing the test current amplitudes by taking the maximum value under each condition, (I/Imax = {1 + exp[(V1/2−V)/k>, as unity. SF, sodium ferulate; Ik, delayed rectifier K^+^ current.

The effect of SF (15.3 μM) on the inactivation kinetic curves of Ik is shown in Fig. 3. The holding potential was set to −70 mV and the currents were induced with a test pulse to +40 mV, during repeated sweeps to +80 mV from a prepulse of −60 mV, in increments of 10 mV. The curves were fitted with the Boltzmann equation (r=0.997). Following the application of 15.3 μM SF, the elicited Ik was inhibited (Fig. 3A) and the values of V1/2 for the inactivation of Ik changed from 6.7±1.8 mV (absence of SF) to 1.2±1.7 mV (n=6, P<0.01), and the slope factor k of the I/Imax curves prior to and following application of SF was −20.7±1.4 and −21.1±1.9 mV, respectively (n=5; P>0.05; Fig. 3B). Compared with the control, SF shifted the inactivation kinetic curve to a negative potential and had no effect on the slope factor.

## Discussion

The identification of novel anti-nociceptive drugs from natural products has been the focus of numerous studies. The effect of ferulic acid-type organic acids following extraction and purification has been of particular interest. A number of papers have reported that ferulic acid acts as an antinociceptive agent (3,17,18); however, whether the K^+^ channel is involved in the anti-nociceptive mechanism remains to be elucidated. As important physiological regulators, Kv channels regulate the action potential shape, membrane potentials and firing adaptation in excitable tissues, including nociceptive sensory neurons (4,5,19,20). K^+^ channel alterations may reflect the clinical antinociceptive action. A number of studies have shown that voltage-gated K^+^ channels have a major role in the regulation of neuronal excitability (21,22).

In the present study, the effect of SF on voltage-gated K^+^ channels in PC12 cells was analyzed using the automated patch-clamp method. The results indicate that SF treatment decreases the Ik of PC12 cells, which suggests that the voltage-activated delayed rectifier K^+^ channel was inhibited following SF treatment.

The analysis of the activation and inactivation kinetic curves of Ik demonstrated that the SF affects the kinetics of activation and inactivation. The activation kinetic curve of Ik shifted to positive potentials and the value of V1/2 increased following the application of SF, which indicates that the activation of the K^+^ channels was delayed. The inactivation kinetic curve of Ik shifted to negative potentials and the value of V1/2 decreased following application of SF. It is known that the inactivation of voltage-activated delayed rectifier K^+^ channels occurs during the process of membrane repolarization in which the Ik, an outward current, decreases gradually during the membrane repolarization process (the voltage value decreases gradually from high to low). Therefore, by analyzing the inactivation kinetic curves of Ik, the reduction in the V1/2 following the application of SF could be explained by the fact that the inactivation of the K^+^ channels was delayed. The delayed activation kinetics may be the major cause for the decrease of Ik following SF treatment. The delayed activation kinetics and inactivation kinetics of Ik may be involved in the analgesic effect of SF, which suggests that the change resulted from the function of the K^+^ channel or its properties.

Generally, it is thought that the inhibition of potassium currents leads to membrane excitation; however, certain studies have reported that Ik is inhibited following anesthetic and analgesic drug application (23,24). It has been hypothesized that excitation of neurons results in overall depression of potassium currents. It has also been suggested that the neuronal depression or excitation caused by inhibition of the K^+^ channel is determined by the location of the K^+^ channel within a neuronal network, which may also modulate the antinociceptive response (23). In the present study, it was found that SF shifted the activation kinetic curve of the delayed rectifier K^+^ channel to higher membrane potentials and the inactivation kinetic curve of the delayed rectifier K^+^ channel to lower membrane potential. This indicates that the delayed activation kinetics result in a delayed process of repolarization, and the delayed inactivation kinetics may result in an increase of K^+^ efflux from the cell, which may be associated with the cell membrane hyperpolarization and lead to neuronal depression. This may help to explain the association between the anti-nociceptive effect of SF and the inhibition of delayed rectifier K^+^ channels.

The association between the inhibition of the delayed rectifier K^+^ channel and the anti-nociceptive effect of SF requires further investigation. Furthermore, as a result of the complicated association between the K^+^ channel, the Ca^2+^ channel and the Na^+^ channel (25,26), the effects of SF on the Ca^2+^ channel, the Na^+^ channel and their associations also require further investigation, which is likely to explain the mechanism of the anti-nociceptive effect of SF. In conclusion, SF treatment significantly inhibits the delayed rectifier K^+^ currents of PC12 cells in a concentration-dependent manner. The mechanism may be associated with the delayed activation and inactivation of the Ik channel.
